# Skin Tone Analysis for Representation in Educational Materials (STAR-ED) using machine learning

**DOI:** 10.1038/s41746-023-00881-0

**Published:** 2023-08-18

**Authors:** Girmaw Abebe Tadesse, Celia Cintas, Kush R. Varshney, Peter Staar, Chinyere Agunwa, Skyler Speakman, Justin Jia, Elizabeth E. Bailey, Ademide Adelekun, Jules B. Lipoff, Ginikanwa Onyekaba, Jenna C. Lester, Veronica Rotemberg, James Zou, Roxana Daneshjou

**Affiliations:** 1 IBM Research – Africa, Nairobi, Kenya; 2 IBM Research – T. J. Watson, New York, NY USA; 3 IBM Research – Europe, Zurich, Switzerland; 4 Stanford University, Stanford, CA USA; 5 University of Pennsylvania, Philadelphia, PA USA; 6 Department of Dermatology, Temple Medical School, Philadelphia, PA USA; 7 University of California San Francisco, San Franciscoa, CA USA; 8 Memorial Sloan-Kettering Cancer Center, New York, NY USA

**Keywords:** Medical research, Medical imaging

## Abstract

Images depicting dark skin tones are significantly underrepresented in the educational materials used to teach primary care physicians and dermatologists to recognize skin diseases. This could contribute to disparities in skin disease diagnosis across different racial groups. Previously, domain experts have manually assessed textbooks to estimate the diversity in skin images. Manual assessment does not scale to many educational materials and introduces human errors. To automate this process, we present the Skin Tone Analysis for Representation in EDucational materials (STAR-ED) framework, which assesses skin tone representation in medical education materials using machine learning. Given a document (e.g., a textbook in .pdf), STAR-ED applies content parsing to extract text, images, and table entities in a structured format. Next, it identifies images containing skin, segments the skin-containing portions of those images, and estimates the skin tone using machine learning. STAR-ED was developed using the Fitzpatrick17k dataset. We then externally tested STAR-ED on four commonly used medical textbooks. Results show strong performance in detecting skin images (0.96 ± 0.02 AUROC and 0.90 ± 0.06 F_1_ score) and classifying skin tones (0.87 ± 0.01 AUROC and 0.91 ± 0.00 F_1_ score). STAR-ED quantifies the imbalanced representation of skin tones in four medical textbooks: brown and black skin tones (Fitzpatrick V-VI) images constitute only 10.5% of all skin images. We envision this technology as a tool for medical educators, publishers, and practitioners to assess skin tone diversity in their educational materials.

## Introduction

Medical textbooks, lecture notes, and published articles used in the curricula of leading medical schools lack adequate representation of skin tones in the images used to demonstrate the manifestations of skin disease^1–3^. For example, a recent manual evaluation of commonly used medical textbooks found significant underrepresentation of Fitzpatrick skin tones (FST) V and VI, which represent brown and black skin tones^1,2^. The COVID-19 pandemic has further highlighted this inequity: manual annotation of published photos of COVID-19 cutaneous manifestations revealed underrepresentation of images depicting dark skin^4^.

Because skin disease appears differently across skin tones, educational materials depicting diverse skin tones are required for a well-trained healthcare workforce^1–5^. Louie and Wilkes suggest that racial inequalities in healthcare (accessibility, delivery and quality) are influenced by the lack of diverse representation in curricular materials^1^. For example, skin cancer diagnoses (e.g., melanoma, squamous cell carcinoma) are significantly delayed in patients of color, leading to increased morbidity and mortality^6^.

Previous analysis of dermatology-related academic materials (journals and textbooks) has shown under-representation of FST V and VI; however, images were annotated and analyzed manually, i.e., a domain expert located each image in a textbook/journal and labeled the skin tone. Unfortunately, this manual approach is not tractable for a large corpus due to its labor-intensive nature, operator visual fatigue, and intra-inter-observer error of skin tone labeling^1,2,4^. *Automatic* skin tone representation assessment using machine learning (ML) promises to significantly aid in identifying bias in medical educational materials and has not been done previously on educational materials.

Machine learning based approaches to skin tone analysis in dermatology have previously been applied only to curated datasets (e.g., ISIC 2018^7^ and SD-198^8^), but not to real world academic materials. One previous approach used individual typology angle (ITA) computed from pixel intensity values^9–11^; the ITA values were then mapped to FST^12^. However previously, a machine learning model trained to classify FST directly from skin images performed better at categorizing FST than ITA-based estimation with conversion to FST^13^. ITA-based methods depend on raw pixel values, making them more sensitive to lighting conditions. These previous models identified that curated skin image datasets used for developing machine learning models in dermatology significantly underrepresented dark skin tones.

**Fig. 1:**
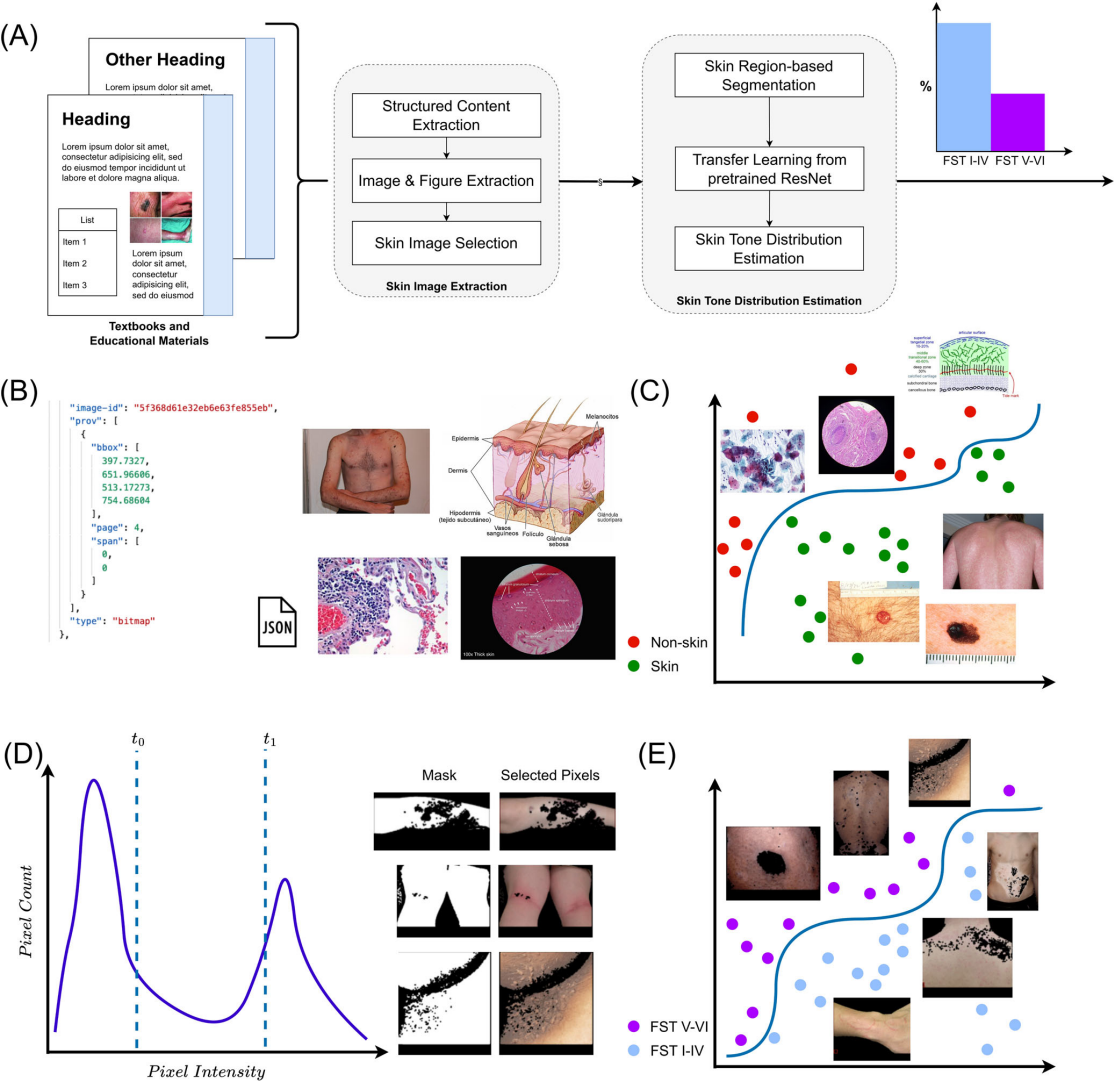
STAR-ED framework overview. **A** STAR-ED framework takes academic materials (e.g., in .pdf format) as input followed by extraction of skin images in the given academic material. Specifically, image pixels that are identified as skin are then utilized to estimate the skin tone category. **B** Corpus Conversion Service (CCS) (7) is an existing document ingestion tool employed to parse different document entities, such as all images and tables in the data. We extracted all images using the JavaScript Object Notation (JSON) (8); output from the ingestion step contains the coordinates and page number of identified images. **C** Since our focus is on images related to skin diseases, non-skin images (e.g., graphical illustrations and pathology images) are discarded using an XGBoost (9) classifier. **D** For each image depicting skin, we masked out non-skin related pixel regions in the foreground and background (e.g., pixels of clothes, laboratory equipment). We employ color-based skin pixel segmentation that extracts pixels that meet a predefined threshold. **E** Finally, the segmented skin regions are fed into a pre-trained deep learning framework, i.e., ResNet^17^ fine-tuned as described in Materials and Methods, to estimate the skin tone category as either light (FST I–IV) or dark (FST V–VI). Images adapted from Wikimedia commons.

In this work, we present the Skin Tone Analysis for Representation in EDucational materials (STAR-ED) framework to automatically assess bias in skin tone representation in medical education materials using machine learning. STAR- ED could be employed on off-the-shelf academic materials, such as textbooks, journals and slides in different file formats (e.g., .pdf, .pptx, .docx). Domain experts (e.g., medical school professors, clinicians) can directly use the outputs to analyze their materials and identify potential biases in representation. The overview of the STAR-ED pipeline is shown in Fig. 1, and it is designed to take academic materials as input and provides a quantification of FST I-IV versus FST V-VI images, automating a task that was previously done manually^2,14^. The main components of the pipeline are automatic *ingestion* of traditional academic materials (textbooks in .pdf format), *parsing* of different entities (figures), *extraction* of images, *selection* of skin images, *masking* of non-skin pixels, and *estimation* of skin tones.

## Results

### Overall pipeline

In this section, we describe the results from STAR-ED, an end-to-end skin-tone representation analysis framework validated on multiple educational data sources in dermatology. Below results are provided for the components of the framework: *skin image selection*, *skin pixel segmentation* and *skin tone estimation*. We describe and validate each step.

### Skin image selection

**Fig. 2:**
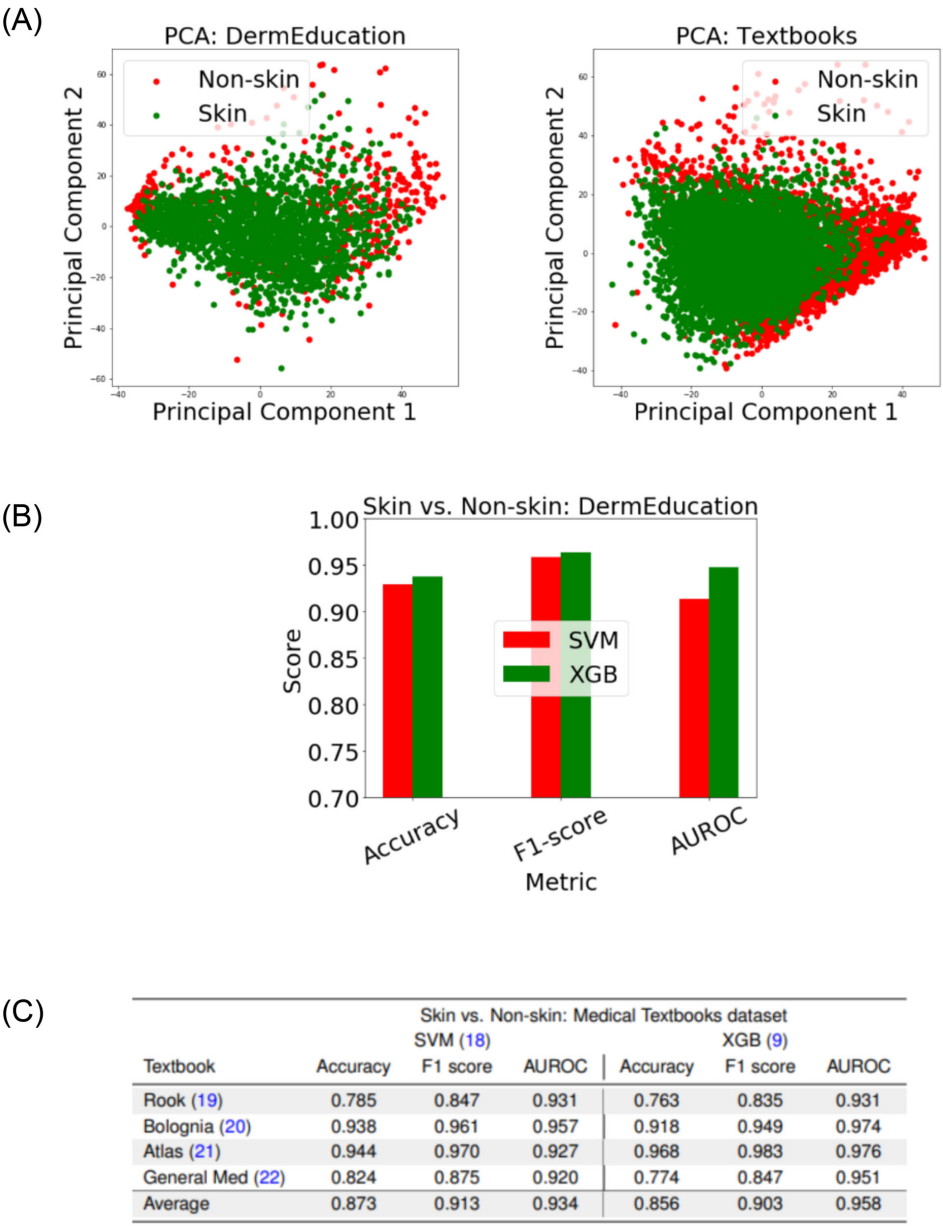
Results for skin image selection step of the STAR-ED framework. Once the images are extracted from the materials, the selection step aims to identify skin images and discards non-skin images (e.g., pathology images). To this end, we extracted a set of features: Histogram of Oriented Gradient (HoG) (23) and mean and standard deviations of image channels in CIELAB (24) color space. **A** This shows the Principal Component Analysis (PCA) visualizations of skin (green) and non-skin (red) images in the two datasets (DermEducation and Medical Textbooks) used for the validation of the selection step. Legend: Red dot – Non-skin; Green ddot - Skin. **B** This demonstrates encouraging performance in identifying skin images in DermEducation using Support Vector Machines (SVM) (18) and Extreme Gradient Boosting (XGBoost) (9) classifiers in a five-fold stratified cross-validation setting. Legend: Red bar – SVM; Green bar – XGB. **C** It shows the comparative performance of these two classifiers when they are used in four dermatology textbooks as an external test. The overall results confirm the benefit of machine learning approaches to identify skin images, and competitive performance is achieved between SVM and XGB classifiers while the latter has a slight advantage and is used in STAR-ED.

To visualize the difference in skin images from non-skin ones, we apply a principal component analysis on the features space that includes Histogram of Oriented Gradient (HoG) and basic statistics (mean and standard deviations) of image channels in CIE LAB color space. The distributions of skin and non-skin images (projected with the two main principal components) are shown in Fig. 2A for both DermEducation and Medical Textbooks datasets (described in Materials and Methods). The skin and non-skin images show substantial overlap, as visualized in the PCA plot. This suggests that single image statistics cannot reliably distinguish between skin and non-skin and motivated us to use machine learning approaches for STAR-ED.

Two classifiers—support vector machine (SVM)^15^ and XGBoost (XGB)^16^—were trained and tested for the skin image selection task. For computing performance metrics, the images containing skin were treated as the positive class and images not containing any skin images were treated as the negative class.

Figure 2B shows the performance of SVM and XGB in the DermEducation dataset using a five-fold stratified cross-validation setting. With the DermEducation dataset, both classifiers achieve competitive performance with XGB resulting in slightly better performance with 0.96 ± 0.008 average F1 score and 0.95 ± 0.013 average area under receiver operating characteristic (AUROC). Figure 2C shows the results when these trained models are validated with the external Medical Textbooks dataset comprised of images extracted by CCS from four medical textbooks. Consistently encouraging performance (>0.9 AUROC) is achieved between the classifiers across the four textbooks confirming the robustness of the framework. Specifically, XGB classifier results in an average AUROC of 0.96 ± 0.02 and F_1_ score of 0.90 ± 0.06 F_1_ across the textbooks. To summarize, skin image detection could be done satisfactorily using traditional machine learning classifiers (without sophisticated deep networks). XGB was used for the final STAR-ED pipeline due to its slightly better performance, particularly in its AUROC, which, unlike accuracy, is independent of single prediction thresholds.

### Skin pixel segmentation

**Fig. 3:**
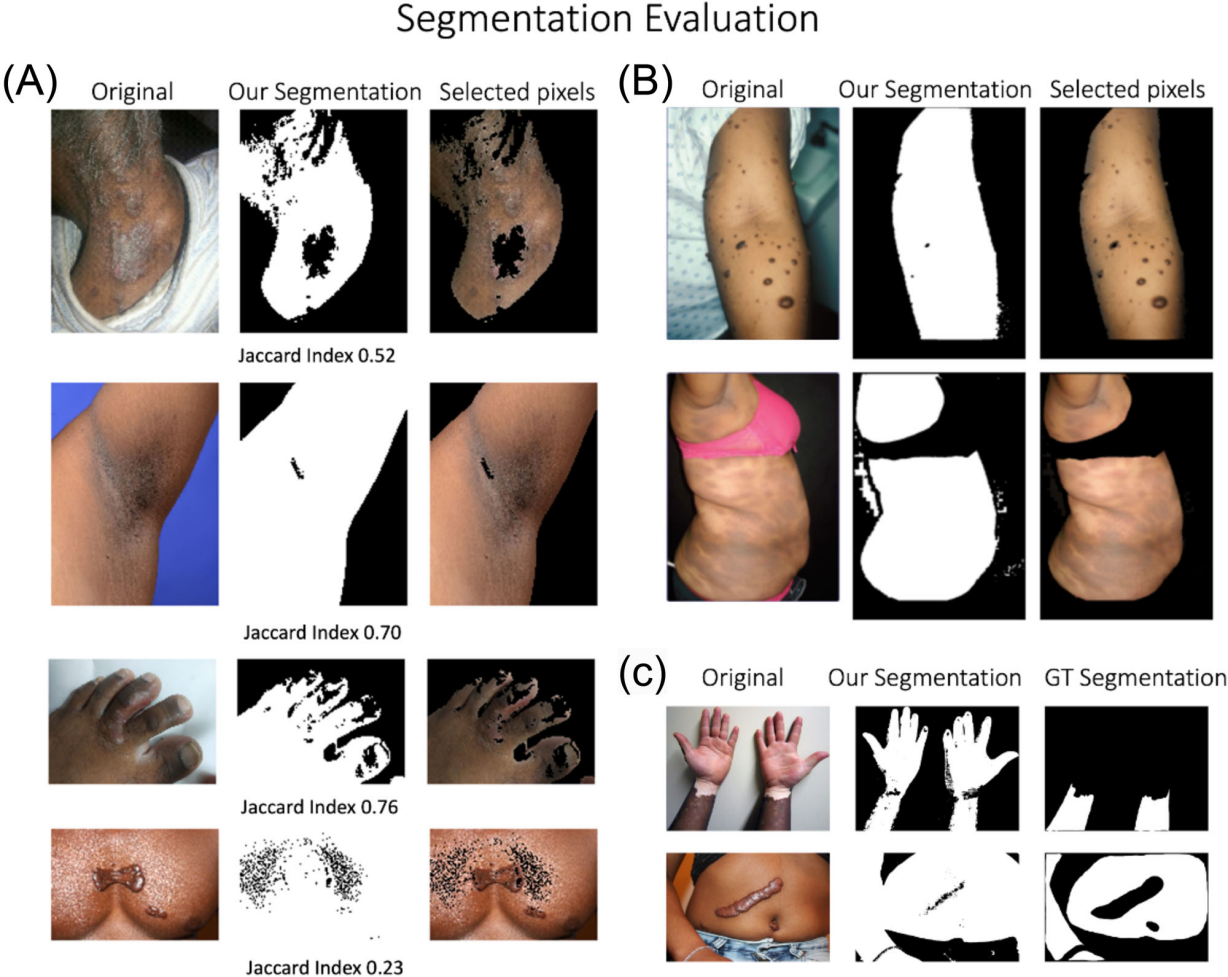
Segmentation of skin. **A** Segmentation of skin pixels examples with their corresponding Jaccard scores. **B** Examples of segmentation of skin pixels, where foreground and background non-skin and lesion pixels are masked. **C** Examples of segmentation comparisons. The first example demonstrates higher agreement between two annotations whereas the second example reflects less agreement between these annotations. Images adapted from Wikimedia commons.

Segmentation of skin pixels aimed to mask out non-skin pixels (e.g., background, foreground) as shown in Fig. 3.

We used the SkinSegmentation dataset (described in the Materials and Methods section) to compare the segmentation results from the proposed method with manual segmentations by a domain expert. Note that the expert segmentations exclude both non-skin pixels and regions containing skin lesions, while the automatic and intensity-based segmentation does not exclude skin lesions at the current stage. Figure 3C shows two examples where maximum and minimum agreement is achieved. Further comparison metrics, such as the Jaccard index is given in Fig. 3A. Overall, the comparison of the segmentation results provides an average false positive rate of 0.24, false negative rate of 0.05, true positive rate of 0.36, true negative rate of 0.34, Jaccard index of 0.51, and accuracy of 0.70. Note that skin-related pixels are treated as the positive class and non-skin related pixels in a skin image (e.g., cloth) are labeled as the negative class in the computation of true positive and negative rates.

### Skin tone estimation

Table 1 shows estimation results (mean and standard deviation) in the Fitzpatrick17K dataset^11^, based on a stratified five-fold cross-validation across multiple machine learning approaches. In the table, we show results on methods when using the raw masked pixels compared to using input engineered features based on HOG + ITA (see Methods section for more details). We categorized the skin tones as FST I–IV and FST V-VI. FST V–VI is labeled as the positive class, and FST I-IV is labeled as the negative class in the computation of precision, accuracy, and F_1_ score metrics. We use weighted metrics to account for class imbalance by computing the average of binary metrics in which each class’s score is weighted by its presence in the true data sample.

**Table 1:** Skin tone estimation performance across multiple machine learning (ML) models and preprocessing techniques.

	Raw Masked Pixels				Feature Vectors (HOG + ITA)			
Model	Accuracy	F_1_ score	Precision	AUROC	Accuracy	F_1_ score	Precision	AUROC
Random Forest^34^	0.87 ± 0.00	0.83 ± 0.00	0.84 ± 0.00	0.77 ± 0.01	0.88 ± 0.00	0.85 ± 0.00	0.86 ± 0.00	0.84 ± 0.00
Balanced Random Forest	0.76 ± 0.00	0.79 ± 0.00	0.86 ± 0.00	0.80 ± 0.00	0.77 ± 0.01	0.80 ± 0.00	0.87 ± 0.00	0.85 ± 0.00
Extremely Randomized Trees^35^	0.87 ± 0.00	0.83 ± 0.00	0.85 ± 0.00	0.80 ± 0.01	0.88 ± 0.00	0.85 ± 0.00	0.86 ± 0.01	0.85 ± 0.01
Ada Boost^36^	0.85 ± 0.00	0.84 ± 0.00	0.82 ± 0.00	0.77 ± 0.00	0.87 ± 0.00	0.86 ± 0.00	0.85 ± 0.00	0.83 ± 0.01
Gradient Boosting	0.86 ± 0.00	0.86 ± 0.00	0.84 ± 0.00	0.77 ± 0.02	0.88 ± 0.00	0.86 ± 0.00	0.86 ± 0.00	0.85 ± 0.01
Pretrained Resnet (STAR-ED)	**0**.**90** ± **0**.**00**	**0**.**91** ± **0**.**00**	**0**.**91** ± **0**.**00**	**0**.**87** ± **0**.**01**	NA	NA	NA	NA

The metrics are based on cross-validation for five stratified folds over the Fitzpatrick17K dataset. Different validations include using *Raw Masked Pixels* without handcrafting features to the ML models. In another validation, features were manually extracted and fed into the ML models. These features include Histogram of Oriented Gradient (*HOG*), which is a commonly employed and simple image representation, and an Individual Topology Angle (*ITA*) that is used to map skin images into Fitzpatrick skin tone categories. Expectedly, traditional models such as Ada boost and Random Forest performed better using handcrafted features, whereas a pretrained ResNet finetuned with Fitzpatrick17^11^ exploited the raw masked pixels due to its capability to learn discriminant features automatically and achieved the highest performance by outperforming all the baseline models.

Bold entries represent the best performing method.

Additionally, we compute the recall metric for all methods. In the pre-trained ResNet-18 (recall = 0.88) we used masked pixels as input. For traditional ML models, we use the Feature Vectors (HOG + ITA), as they show similar performance as pixels while reducing the runtime of the training and testing (See Table 1). We can observe that from traditional methods (Random Forest = 0.61, Extremely Randomized Trees = 0.61, Ada Boost = 0.64, and Gradient Boosting = 0.65), the Balanced Random Forest (recall = 0.77) achieves the best recall for both skin tones, while the other methods perform poorly for FST V-VI classification.

We found that the weighted ResNet-18^17^ deep learning framework, pretrained with ImageNet^18^, which contains 11,689,512 parameters, and finetuned with Fitzpatrick17K^11^, has the best performance and incorporated this method of skin tone estimation for the STARE-ED framework.

**Fig. 4:**
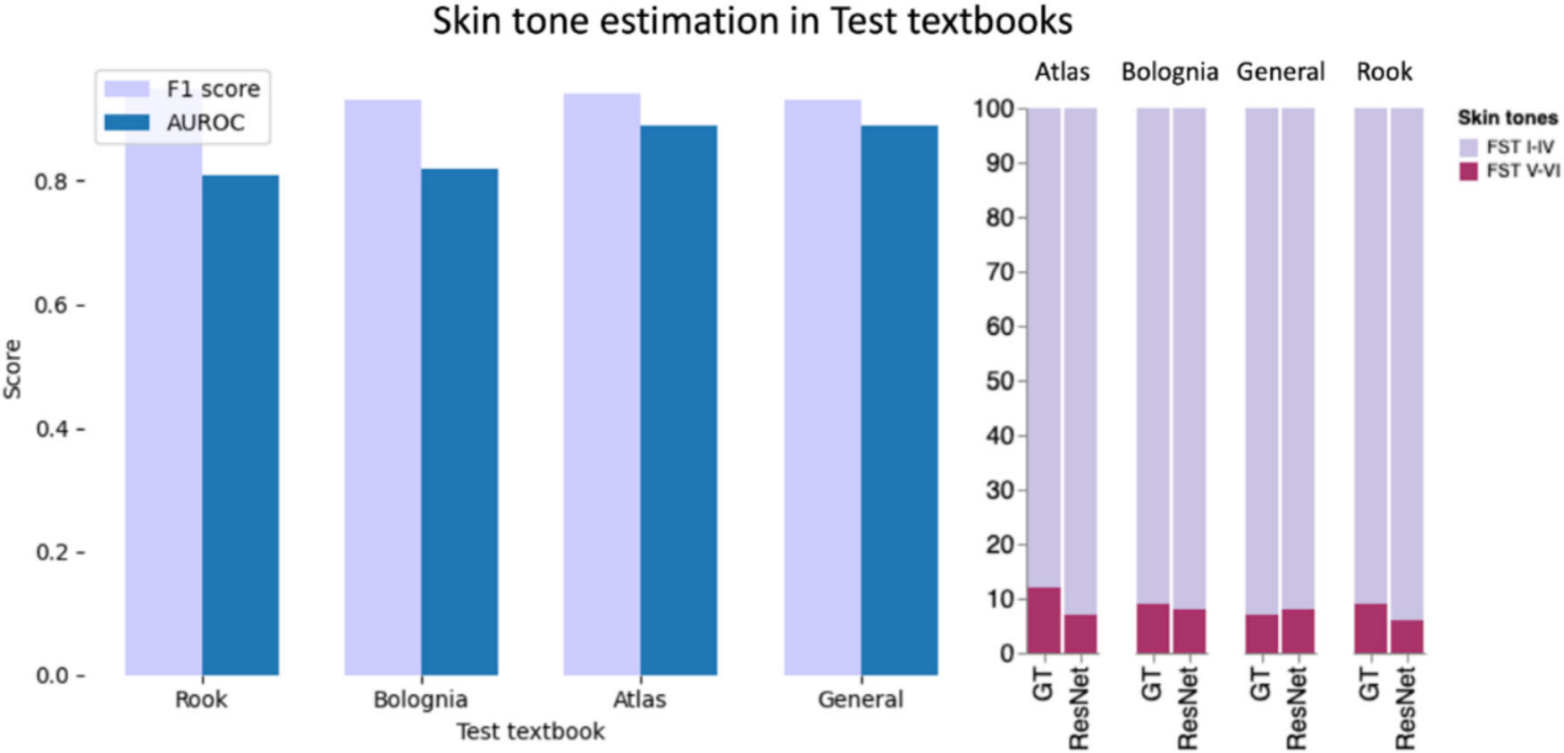
Tone estimation in external validation textbooks. The performance is evaluated using AUROC and F1 score. We can also observe skin tone proportions in each textbook, tones estimated by our proposed method and the ground truth (GT). First bar graph legend: Purple bar – F1 score; Blue bar – AUROC. Second bar graph legend: Purple bar: FST I-IV skin tones; Magenta bar: FST V-VI skin tones.

After training and validation in the Fitzpatrick17k dataset, we evaluated the skin tone estimation approach using multiple external sources. See Fig. 4 for AUROC and F_1_ scores for each of the four textbooks in the Medical Textbooks dataset using a pre-trained ResNet-18 finetuned as described in Materials and Methods. Figure 4 also shows the proportion of FST I–IV versus FST V–VI images for each textbook as estimated by STAR-ED and compared to the ground truth. We observe in each dermatology textbook used for STAR-ED validation, there is an under-representation of FST V–VI, in all cases lower or equal to 10.5%. Previously, these textbook images were hand-labeled to assess for bias in skin tone representation in a process that took over 100 person–hours compared to the STAR-ED framework, which generates a bias assessment within minutes^2^.

We perform additional external testing with DermEducation, a standalone image dataset used by dermatologists to study for board exams (see Materials and Methods section). When a weighted ResNet^17^ deep learning framework pretrained with ImageNet^18^ and finetuned as described in Materials and Methods is used, we obtain an AUROC of 0.87 and an F_1_ score of 0.91 for skin tone estimation compared to other established methods, such as balanced trees^19^ with AUROC of 0.82 and F_1_ score of 0.80. Evaluation of ITA-based Fitzpatrick index mapping (see Supplementary Table 1); results in the lowest skin tone estimation performance with F_1_ score 0.36.

We find that STAR-ED demonstrates in an automatic manner a clear bias in representation across dermatology educational materials, and textbooks for skin tone FST V-VI (≤10.5%).

## Discussion

Disparities in dermatological diagnosis may be related to inequities in dermatological education materials. Particularly there are consistent reports by domain experts on the lack of FST V-VI images in the materials used to train dermatologists and primary care physicians^1,2^. Thus far, efforts to understand representation biases in these materials have been done manually, which is labor-intensive and impractical for large-scale applications. The contribution of this paper is the development and validation of an end-to-end machine learning tool, STAR-ED, that automatically ingests these materials and provides representation analysis can facilitate detection and understanding of representation bias. Such a tool could be impactful by providing first-hand awareness of potential bias prior to publication or quickly post-publication. STAR-ED is flexible to work on different formats of educational materials, e.g., *.pdf*, scanned books as images, slides in *.pptx* and word documents in *.docx*. Thus, STAR-ED could be used beyond just textbooks, and could also assess research papers, image study sets, and lecture slides.

To build STAR-ED, we tested various machine learning methods in order to build an end-to-end workflow that performed skin image selection, skin-pixel segmentation, and skin tone estimation. A challenge of estimating skin tone distribution from complex materials such as textbooks is parsing and identifying skin images from other materials (e.g, text, tables). A number of rule-based methodologies for ingesting documents have been previously developed; however Staar et al developed a machine learning based approach which allows greater flexibility across document types^20^. However, unlike a curated dermatology dataset, images extracted from textbooks or other educational materials often contain non-skin images as well. To distinguish between skin and non-skin images, we created a feature vector for each image that included the histogram of oriented gradient (HOG) descriptor and an intensity-based feature based on the CIE LAB color space. Previously, HOG descriptors have been used for distinguishing skin lesions^21^. Additionally, previous work on separating skin and non-skin images have relied on clustering in the color space; a comparison of normalized RGB, HSV, YCbCr, CIE LAB, and CIE Luv color spaces for building probabilistic classifiers to identify skin found the CIE LAB had the best performance^22^. We combine these features and find that the XGB classifier had good and scalable performance in separating skin from non-skin images. Images of skin often have foreground and background objects, requiring the identification of regions of the image that display skin. For skin pixel segmentation, our current methodology utilizes an intensity-based skin pixel segmentation technique. Previous work on the International Skin Imaging Collaboration (ISIC) data used mask R-CNN for skin lesion segmentation; however, ISIC images are dermoscopic, which are more standardized than the heterogeneous clinical images seen in textbooks^23^. Moreover, our simplified approach allows a more lightweight model for widespread application of this framework while still allowing downstream skin tone prediction that is close to the ground truth (Fig. 4). Finally, we tested multiple different methodologies for skin tone assessment and found that a pretrained Resnet model finetuned on the Fitzpatrick17k dataset had the best performance for predicting FST I-IV and FST V-VI images. We validated the entire pipeline across four textbooks showing skin disease; these textbooks were selected due to their previous identification as core dermatology textbooks in prior work^2^.

We were able to use STAR-ED to recapitulate the findings in the literature, which shows significant underrepresentation of FST V-VI skin in dermatology educational materials. STAR-ED allows this bias assessment to occur at scale and without the need for hours spent labeling manually. We envision STAR-ED allowing medical educators, publishers, and practitioners to quickly assess their educational materials.

Future work aims to pilot STAR-ED among different publishers and content creators around the world. We envision this technology as a tool for dermatology educators, publishers and practitioners to quickly assess their educational materials, which could be scaled to other domains (e.g., history) to automatically identify lack of diverse representation.

While encouraging performance is achieved in detecting skin images and estimating the skin tone categories, the proposed pipeline does not consider non-image content of a given academic material, e.g., texts, authors list and tables, which could be later integrated to provide multi-modal representation analysis.

A limitation of our skin pixel segmentation methodology is that it does not fully exclude diseased or lesional skin, which may have pigmentation patterns that do not represent the appearance of the individual’s healthy skin. Future iterations of STAR-ED will aim to add a step that segments diseased or lesional skin for added granularity. For skin tone estimation, we separated images to two groups: FST I-IV and FST V-VI in order to capture the lack of brown and black skin tones in educational materials. This model was built to manually recapitulate numerous prior studies in the space of educational material bias, which have focused on FST V and VI^2^. While this means we do not capture further granularity in skin tones, it does assess the most historically excluded skin tones. Skin tone assessment from images alone is also limited by differences in color balancing across different cameras and differences in lighting, both of which can affect the appearance of skin^23^. However recent literature has shown that the most accurate labeling occurs with adjacent skin tones, such FST V and VI^24^. Moreover, we used trained non-experts for labeling ground truth skin tone, but were able to validate against a subset of domain expert-labeled images. Recent work has shown that trained non-experts can perform similarly to expert labelers, especially in light of the variability seen even among experts^24^. While we used the Fitzpatrick skin tone scale for labeling skin tone, this scale has its own biases and subjectivity; dermatologists have discussed the merits of using alternative scales for skin tone estimation^25^. Future iterations of this work could incorporate any alternative skin tone estimation scale that is developed.

## Methods

### Pipeline development

In this section, we describe the datasets used for training and testing our framework and the machine learning algorithms used. This study was IRB exempt due to the use of publicly available data.

### Datasets

The description of how each dataset is used during methods development is described in Supplementary Fig. 3. DermEducation is a convenience image set of dermatology images used for educational purposes. DermEducation contains containing 2708 total images, among which 461 are non-skin images, 2247 skin images (1932 FST I-IV and 315 FST V-VI). DermEducation was used to train the skin versus non-skin classifier. Additionally, it was used to validate the proposed skin tone estimation by comparing it with ITA-based tone estimation. Labeling of skin versus non-skin and skin tone was done by a medical student and reviewed by a dermatologist for accuracy.

The SegmentedSkin dataset is a convenience image set of open source dermatology images selected by a dermatologist from Wikimedia. A dermatologist created segmentation masks of healthy skin for these 22 images. This dataset was used to validate skin pixel segmentation.

Fitzpatrick17K^11^ is a publicly available dataset with 16,577 clinical images sourced from two online open-source dermatology atlases with FST labels generated by dermatologists previously. After preprocessing we used 13,844 images depicting FST I-IV and 2168 images depicting FST V-VI. Fitzpatrick17K was used to train and validate our skin tone estimator.

**Fig. 5:**
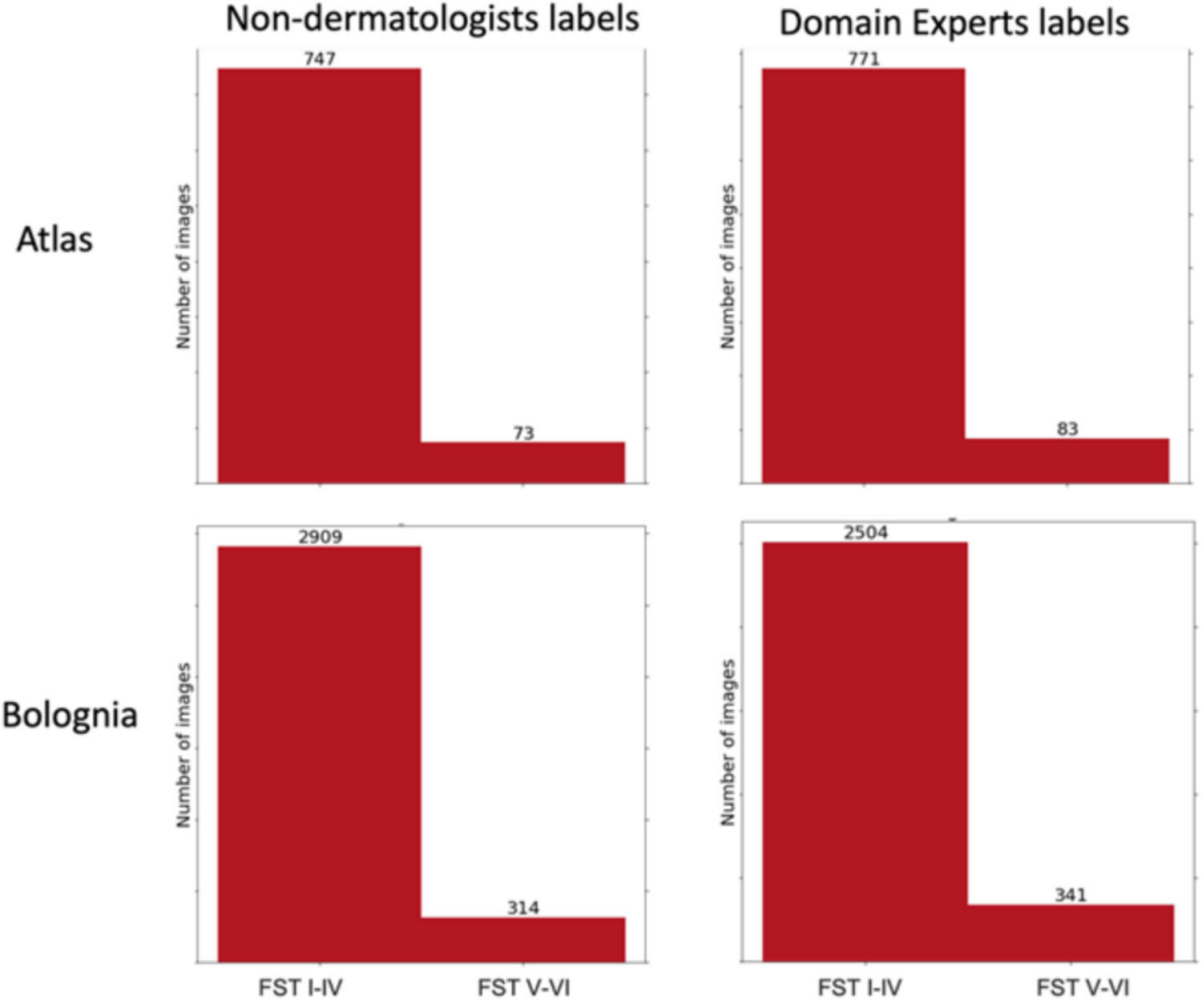
Domain expert versus non-dermatologist labels. Label distributions from non-dermatologists and previously reported total numbers by domain experts across multiple chapters from Bolognia and Atlas books.

For additional external testing and to demonstrate how our framework can be used on real world educational materials, we also used four medical textbooks personally owned by the authors. As a group, we refer to this as the Medical Textbooks dataset, which is comprised of: Rook’s textbook of dermatology^26^, Bolognia 4e^27^, Fitzpatrick Color Atlas 8e^28^, and Fitzpatrick Dermatology in General Med 9e^29^. After using the corpus conversion service to extract images, we filtered out tiny images (<100 pixels in any dimension). See Table 2 for a summary of the datasets used in this paper: Medical Textbooks (containing four textbooks), DermEducation and publicly available Fitzpatrick17K. Note that proportion of skin images to non-skin images vary across the textbooks and the datasets. For example, Atlas^28^ has 822 skin images and only 57 are non-skin images; on the other hand, Fitzpatrick General^29^ has only 1881 skin images compared to 1096 non-skin images. For Medical Textbooks dataset, images were manually labeled as skin versus non-skin by the authors . Skin images were labeled as FST I-IV and FST V-VI by non-dermatologists who were trained on previous examples. The label distributions were compared to those previously reported by domain experts on a subset of images and found to be similar, see Fig. 5. The level of agreement between a subset of images labeled both by domain experts and our trained labelers was 0.887 for Fitzpatrick, 0.860 for Atlas, and 0.855 for Bolognia (Fig. 5). The labels for DermEducation were done by a medical student, whereas Fitzpatrick17k labels were included with the dataset^11^.

**Table 2:** Details of the datasets used in this work.

Book	Chapters	Images	Skin images	FST V-VI	FST I-IV
Rook^26^	3	553	343	21	319
Bolognia^27^	160	4150	3225	311	2853
Atlas^28^	36	879	822	73	747
Fitzpatrick^29^	217	2977	1881	221	1620
DermEducation	NA	2708	2247	315	1932
Fitzpatrick17K^11^	NA	16,577	16,012	2168	13,844

Across the two datasets (and four textbooks), the ratio of FST V-VI images to FST I-IV images is significantly small reflecting the severe imbalance in representations of skin tones.

*NA* not applicable.

### Machine learning pipeline

The overview of the proposed method is shown in Fig. 1. We describe the main components of the proposed pipeline below.

### Document ingestion

We used the Corpus Conversion Service (CCS) to ingest academic materials in a scanned and programmatic PDF document format^20^. The CCS is a cloud based service, which can ingest large corpora at scale. It uses AI models^30^ to convert PDF documents into structured text-files in JavaScript Object Notation^31^. In addition to extracting the main-text from the documents, the CCS also allows the user to easily identify the tables and images with their captions and their position in the documents. This image-extraction capability facilitates the extraction of images that can be used as (raw) data for the work described in this paper.

### Skin image detection

In order to achieve simplicity in the step of detecting skin images, we use histogram of oriented gradient (HOG) descriptor, which is commonly used in object detection and is invariant to local geometric or photometric transformations^32^. The HOG feature vector for an image I, (**h**_*i*_) is computed from magnitude weighted histogram of directions bins obtained from the gradient of the pixel intensity values in the horizontal (*G*_*x*_) and vertical (*G*_*y*_) directions. *G*_*x*_(*r, c*) = *I*(*r, c* + 1) − *I*(*r, c* − 1) and *G*_*y*_(*r, c*) = *I*(*r* + 1*, c*) *−* *I*(*r* − 1*, c*) represent the gradients of the pixel identified by the *r*th row and *c*th column. The angle related to these gradients is obtained as *θ*_*i*_(*r, c*) = arctan(*G*_*y*_*/G*_*x*_) and its magnitude is defined as *M*_*i*_(*r, c*) = ^p^*G*^2^_*y*_ + *G*_*x*_^2^. The angle values are binned to *C* = 32 clusters following a sensitivity analysis across a range of bins, and each *θ*_*i*_ value is mapped to the closest cluster weighted by the corresponding magnitude *M*_*i*_. Furthermore, we added direct pixel intensity values after the RGB color space is transformed to CIE LAB color space (i.e., *L*, *a* and *b* channels) which is known to be robust across different imaging devices. The feature vector derived from these channels in the image I is **p**_*i*_ = [*µ*_*L*_,*µ*_*a*_,*µ*_*b*_,*σ*_*L*_,*σ*_*a*_,*σ*_*b*_], where *µ* represents the mean value and *σ* represents the standard deviation value. The overall feature vector is the concatenation of the HOG features (**h**_*i*_) and the intensity-based features (**p**_*i*_), resulting in a 38-dimensional final feature vector for skin image detection.

The classification stage is validated using both SVM^15^ and XGBoost^16^ algorithm, and the train-test strategy uses five-fold stratified cross validation with the DermEducation dataset. For SVM, we used the RBF kernel as it better encodes the relationship between features in a nonlinear fashion. To this end, we set nu: the parameter that controls the training error (i.e., the number of support vectors) to 0.01 and the gamma parameter determines the influence of radius on the RBF kernel and it was set to 0.05, targeted to avoid overfitting during training. For the XGBoost classifier, we employ a cross validation (cv) based calibration using cv folds = 3, where the hyperparameters are set from the best performing fold. We employ Area Under Operating Receiving Characteristics (AUROC) and F_1_ score as our performance metrics.

### Skin pixel segmentation

There are multiple approaches for skin segmentation. We can classify the strategies as threshold-based, model-based, and region-based methods. Saxen and Al-Hamadi^33^ showed that region-based are the best performing methods under color segmentation (no texture information is used/evaluated). As our overall goal is to classify binary skin tones, we opted to use skin segmentation approaches rather than lesion segmentation. When more granularity is needed, we will need to consider lesion pixels and not only the skin vs. non-skin pixels approach. We use a combination of a region-growing algorithm and color-based segmentation in the HSV and YCbCr color spaces for the initial experiments. First, we convert our RGB images to HSV and YCbCr color spaces. The used ranges were based on previous published papers^33^. Second, after we clip the images, we apply watershed and other morphological operations.

### Skin tone estimation

To perform skin-tone classification, we use the Fitzpatrick17k dataset for training and evaluation using cross-validation. For external testing we used the textbooks detailed in Table 2 and DermEducation. As input data we only use the skin pixels extracted from our previous section (See Selected pixels in Fig. 3B, C). We aimed to label skin images as FST I-IV or FST V-VI. For these experiments, we explored feature-engineered and deep learning approaches. For the feature-engineered vectors, we use the concatenation of the HOG feature vector, the mean and standard deviation of Luminance (L) and Yellow (b) channels in CIE LAB color space and ITA values, which are highly correlated to melanin indexes^9,12,23^. These feature vectors were used in multiple Ensemble methods (Random Forest^34^, Extremely Randomized Trees^35^, AdaBoost^36^ and Gradient Boost^16^), see Table 1 as all models performed similarly at an average level. Random Forest and Randomized trees performed similarly to the other methods, requiring less compute time than Ada Boost and Gradient Boosting. All the models were implemented with scikit-learn v0.24.2^37^ and imbalanced-learn^38^. Additionally, we evaluated deep learning models. We used a pretrained ResNet-18, which is a convolutional neural network that is 18 layers deep. The pretrained weights contain 11689512 parameters. The network was trained on more than a million images from the ImageNet dataset^18^. After loading the weights, we modify the last layer to consider only two classes (FST I-IV and FST V-VI) and perform a weighted retraining for twenty epochs. The retraining was performed with standard Stochastic Gradient Descent optimization on weighted cross-entropy loss, a learning rate of 1*e*^*−*3^ with a linear decay, and a batch size of 32. The implementation was done with the Scientific Python Stack v3.6.9^39^ and Pytorch v1.8.1^40^. Results can be seen in Table 1 and Fig. 4. We also tested an existing approach that maps ITA values on to Fitzpatrick skin tone. When using ITA-based methods, the ITA is later mapped to FST as shown in Supplementary Table 1.

The six Fitzpatrick skin tone indices are then merged into two categories (FST I-IV and FST V-VI) and results are compared with STAR-ED. The skin tone estimation was evaluated across all methods with a data split of 70% of the data used for training, 10% for validation, and 20% for training. These splits only apply to the Fitzpatrick17K dataset; the rest of the datasets were used purely as testing datasets.

### Reporting summary

Further information on research design is available in the Nature Research Reporting Summary linked to this article.

### Supplementary information

Supplementary Information

Reporting Summary

## Data Availability

DermEducation is a private educational dataset that is available on request. The textbooks consisted of Rook’s textbook of dermatology (ISBN 9781118441190), Bolognia 4e^27^ (ISBN 9781118441190), Fitzpatrick Color Atlas 8e (ISBN 9781259642197) and Fitzpatrick Dermatology in General Med 9e (ISBN 9781259642197). Fitzpatrick17k is publicly available at https://github.com/mattgroh/fitzpatrick17k.

## Code availability

Code for the skin image detection, skin pixel segmentation, and skin tone estimation and the SkinSegmentation dataset is available here: https://github.com/IBM/star-ed.
