# Supplementary material for: Skin Tone Analysis for Representation in Educational Materials (STAR-ED) using machine learning
Source: NPJ Digit Med. 2023 Aug 18;6:151. doi: 10.1038/s41746-023-00881-0 (PMC10439178; doi:10.1038/s41746-023-00881-0)
Supplement: Supplementary file 1 — Supplementary Information [file 41746_2023_881_MOESM1_ESM.pdf]

Supporting Information

ITA-based methods

When using ITA-based methods, the ITA is later mapped to FST as shown in (Table S1). We find that mapping ITA to FST performs worse than predicting FST directly. For example, with the DermEducation dataset, ITA-based skin tone evaluation results in many FST I-IV images being categorized as FST V-VI. The dataset has 315 FST V-VI images, but ITA-based methods categorize 1247 out of 2247 images as FST V-VI.

Supplementary Table 1. Mapping of ITA values to Fitzpatrick skin indices (FST) used in all the experiments and label standard across all datasets. In our binary skin tone analysis FST I-IV are merged into light tone whereas FST V-VI are merged into dark tone.

ITA value	FST
$ITA > 41$	I
$34.5 < ITA \leq 41$	II
$28 < ITA \leq 34.5$	III
$19 < ITA \leq 28$	IV
$10 < ITA \leq 19$	V
$ITA \leq 10$	VI

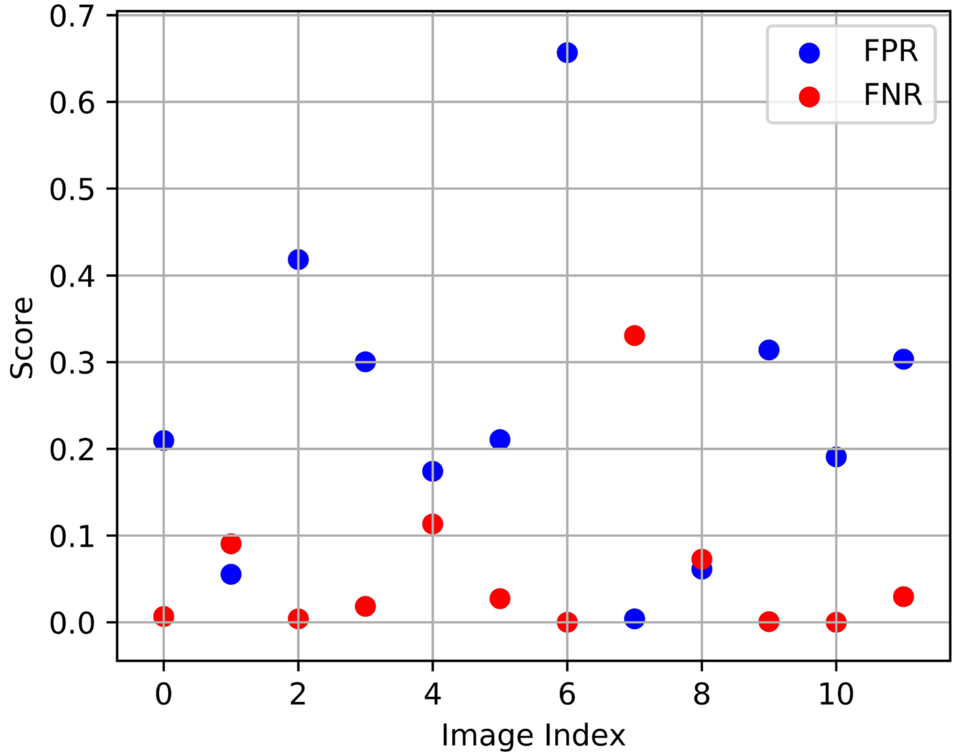

Supplementary Figure 1. Comparison of the false positive rate (FPR) and false negative rate (FNR) between skin pixels segmented using the proposed framework and manual segmentation by domain experts. Twelve skin images were used to evaluate the segmentation outputs. Note that the proposed method resulted in higher FPR compared to FNR. This is partly due to the segmentation of lesion pixels in the manual segmentation, which results in smaller skin regions (pixels) in the manual segmentation.

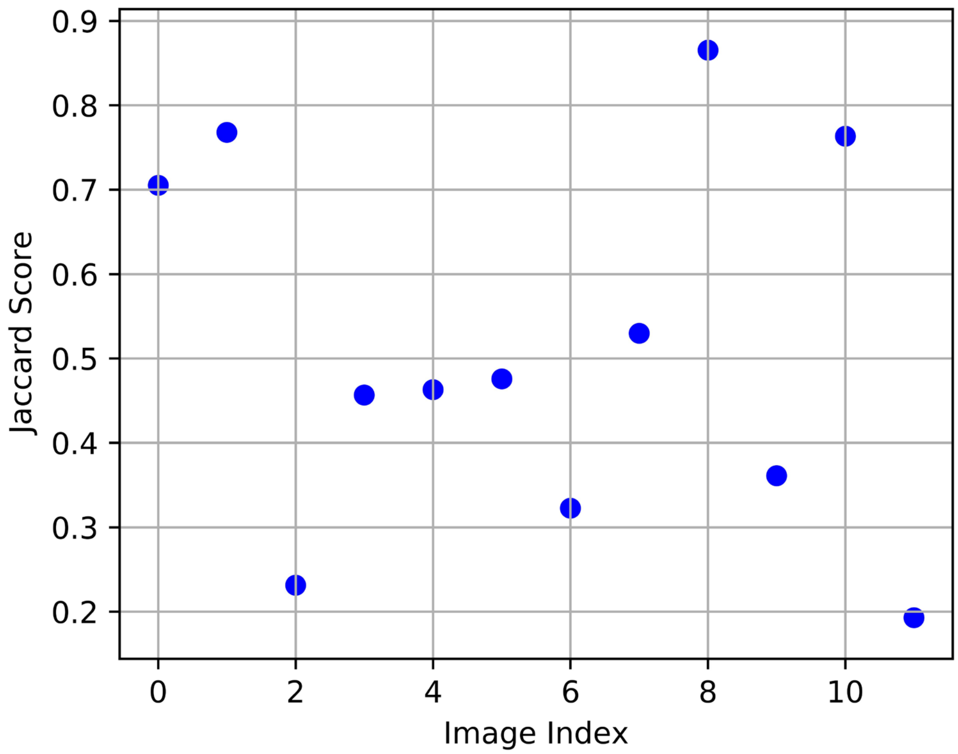

Supplementary Figure 2. Jaccard similariy scores of the segmentation results from the proposed framework and manual segmentation are shown. Segmentation results characterized by higher FPR and/or FNR values in Fig. S1 are shown to demonstrate lower Jaccard similarity.

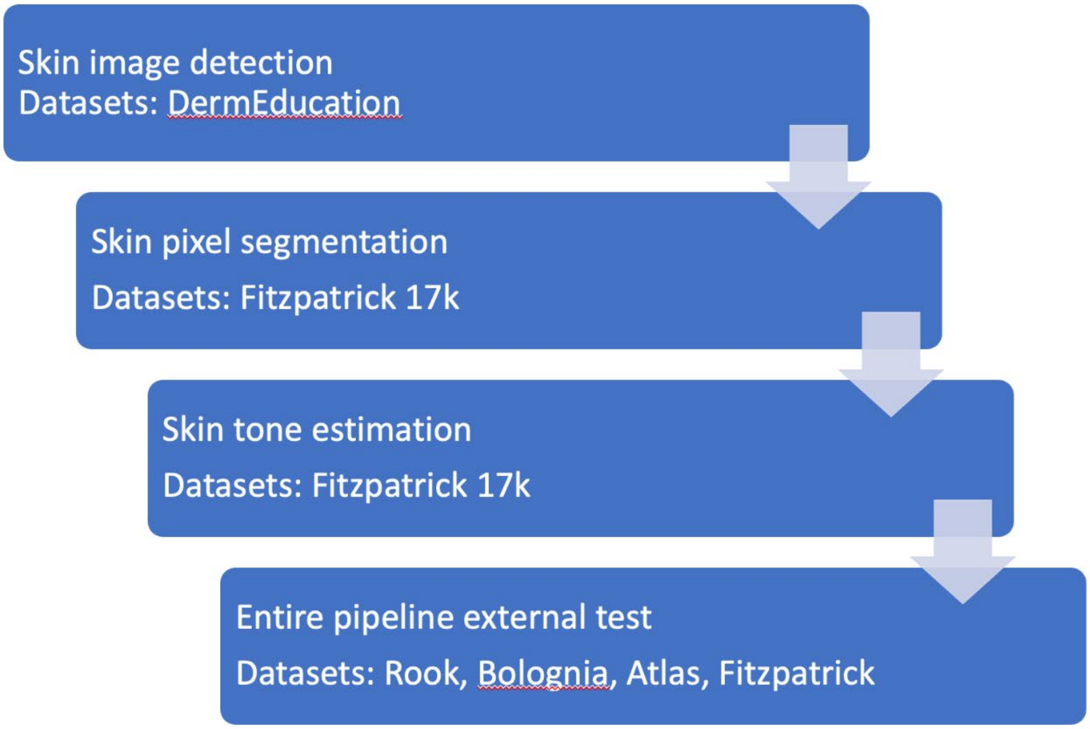

Supplementary Figure 3. Datasets used for each task in the pipeline.
